# How should we prepare for an outbreak of reintroduced live polioviruses?

**DOI:** 10.2217/fvl-2017-0131

**Published:** 2017-01-20

**Authors:** Kimberly M Thompson, Radboud J Duintjer Tebbens

**Affiliations:** 1 Kid Risk, Inc., Orlando, FL 32832, USA; 2 College of Medicine, University of Central Florida, Orlando, FL 32827, USA

**Keywords:** disease outbreaks, dynamic modeling, eradication, polio

Once a terrifying but common infectious disease, polio should soon become an agent only of concern for low-probability but potentially high-consequence reintroduction events. During the polio endgame (i.e., while we complete wild poliovirus eradication, coordinate cessation of all use of the 3 serotypes of live attenuated oral poliovirus vaccine, and contain all live polioviruses in laboratories and other facilities), the risks of polioviruses will still require management. The choices that we make will determine the ultimate success or failure of polio eradication, and we need to remain prepared to manage the endgame and long-term risks.

## Wild poliovirus eradication status at the end of 2016

Although most people think of polio as a single disease, three stable serotypes exist (i.e., 1, 2 and 3). Eradication requires stopping all of them, which in practice means globally interrupting the transmission of three separate viruses. By late 2016, under the leadership of the global polio eradication initiative (GPEI), coun-tries achieved significant progress toward stopping the transmission of all wild polioviruses (WPVs) [1]. With the certification of serotype 2 WPV eradication declared in September 2015 [2], in late April and early May 2016, the GPEI globally coordinated cessation of all serotype 2 containing oral poliovirus (OPV) use [3]. The extensive global polio laboratory network last reported a serotype 3 WPV case in northern Nigeria in November 2012 [4] and reported the lowest ever number of annual paralytic cases caused by serotype 1 WPV in 2016 [1].

## OPV cessation after certification of WPV eradication

Although OPV represents the workhorse vaccine for the GPEI, its use comes with risks [5,6]. OPV induces life-long protection from paralysis through infection, which can spread to susceptible contacts to effectively immunize them. However, in rare instances, individuals experiencing a first infection with OPV may develop vaccine-associated paralytic polio (VAPP) (i.e., ∼ 1 VAPP case per million first OPV infections). This translates into an estimated 400 VAPP cases globally for 2012 [7]. Furthermore, OPV use in populations with low immunization coverage can lead to circulating vaccine-derived polioviruses (cVDPVs). If OPV-related viruses find enough susceptible individuals in a population to circulate, instead of dying out, they lose their attenuating mutations and evolve to become cVDPVs that behave like WPVs [5,8]. Finally, a very small number of individuals with B-cell immunodeficiency exposed to OPV or OPVrelated viruses can excrete an immunodeficiency-associated vaccine-derived poliovirus (iVDPV) long term, which may potentially reintroduce a live poliovirus into the population [5,9]. OPV offers the best tool to stop WPV transmission and much lower risks of paralysis than WPV (i.e., 400–20,000-times lower paralysis-to-infection ratios than for WPV, depending on the serotype) [5,10].

After WPV eradication, the small, nonzero risks of VAPP and VDPVs become unacceptable, particularly given the existence of inactivated poliovirus vaccine (IPV) as an alternative. Largely to avoid VAPP, higherincome countries now use IPV or a sequential IPV/OPV schedule for poliovirus vaccination. IPV induces humoral immunity that protects the vaccine recipient from developing paralysis if subsequently exposed to a live poliovirus, which means an IPV/OPV sequential schedule prevents most VAPP cases. As an inactivated vaccine, IPV does not induce infection, spread secondarily, or cause VAPP or VDPVs. However, IPV costs significantly more to produce and administer than OPV, and its use will not stop poliovirus transmission in a population characterized by high poliovirus transmissibility [10,11]. During the polio endgame, all countries plan to introduce at least one IPV dose into their routine immunization schedules, but implementation of this effort currently remains limited by available IPV vaccine supply [12].

## Managing cVdPV risks

We can prevent cVDPVs from emerging and circulating by maintaining high population immunity to transmission (i.e., the collective ability of all individuals in a population to participate in transmission with or without exhibiting paralytic symptoms) in all populations, which requires achieving and maintaining high immunization coverage as long as OPV use continues. Recognizing the dynamics of evolution at the time of ending OPV use, many countries conducted intensive supplemental immunization activities before globally coordinated cessation of serotype 2 OPV to ensure subsequent die-out of homotypic OPV-related viruses. Countries that rely on supplemental immunization activities should continue to conduct them with bivalent OPV (bOPV, which contains serotypes 1 and 3) to maintain high population immunity to transmission and prevent cVDPVs from circulating after the coordinated stop of bOPV [13]. Because cVDPVs represent highly transmissible and neurovirulent viruses, the GPEI established prerequisites for OPV cessation that included stopping all persistent cVDPVs. Unfortunately, the GPEI discovered evidence of ongoing transmission of a serotype 2 cVDPV in the insecure and access-restricted Borno State of Nigeria only after the globally coordinated switch from trivalent OPV to bOPV. The failure to prevent a cVDPV necessitates a response to the cVDPV outbreak that occurs after OPV cessation by aggressively increasing population immunity to transmission using the monovalent OPV (mOPV) that corresponds to the outbreak virus [14,15]. The GPEI initially responded to the persistent serotype 2 cVDPV in Borno with serotype 2 mOPV, but could not access all areas with the campaigns. Surveillance subsequently also detected ongoing serotype 1 WPV transmission, which shifted the focus to responding to the WPV outbreak with bOPV [16], and decreased the chances of stopping the serotype 2 cVDPV transmission by diverting attention from serotype 2 mOPV campaigns. Inaccessible areas represent a serious threat to the success of polio eradication. Finding ways to access and vaccinate individuals in these areas remains the only way to end all live poliovirus transmission.

## ongoing surveillance

High-quality surveillance must continue during the endgame [17], although maintaining efforts becomes more difficult as the expectations of finding polioviruses decrease. Current poliovirus surveillance depends on obtaining stool samples from patients that present with acute flaccid paralysis. The GPEI increasingly includes the systematic collection and testing of environmental samples to look for polio viruses in sewage in some areas, which might indicate circulation prior to detection of a case. Continued surveillance provides the oppor tunity to identify, respond rapidly, contain, and stop transmission of any reintroduced poli oviruses [10,14], and subsequently to increase confidence in the absence of transmission [17]. The presence of acute flaccid paralysis cases should motivate the continued need to test specimens to rule out poliovirus, and we must remain vigilant.

## Managing iVdPV risks

Assuming we manage the risks well and cVDPVs die out, the relatively very rare iVDPVs could still potentially restart transmission. Efforts to develop polio antiviral compounds to treat iVDPV excretors to clear their infections will create motivation to screen for asymptomatic individuals who may benefit from treatment and protection from VAPP. Screening for and treatment of asymptomatic iVDPV excretors represents a critical element of protecting the population from reintroductions [18].

## Containment

As occurred with smallpox, global risk management efforts will depend on aggressive containment of live polioviruses to minimize the risks of reintroduction from laboratories and vaccine manufacturers.

## Routine immunization with iPV

The USA and other relatively high-income countries will likely continue vaccination with IPV for the foreseeable future, particularly with its increasing delivery in combination vaccines. Since the risks of iVDPVs depend on the survival of immunodeficient patients, which remains longer in relatively higher-income countries [9], the strategy of continuing IPV in routine immunization will mitigate the iVDPV risks [10]. However, at some point relatively lower-income countries may stop including any polio vaccine (including the relatively expensive IPV) in their routine immunization programs, depending on the global minimum policy [6].

## Outbreak response planning & vaccine stockpile

While we continue to manage risks, in addition to other efforts, the world will need stockpiles of mOPV and IPV available to rapidly respond to a live poliovirus outbreak [14]. We will need to ensure aggressive response to a transmissible and neurovirulent live poliovirus, because failing to do so may lead to the need to restart OPV production and use at the global level [14]. In the long term, using mOPV for outbreak response may pose risks associated with reintroducing exported live polioviruses into populations outside of the outbreak area. While IPV may provide sufficient population immunity to stop transmission in some countries, in other countries that are characterized by significant fecal-oral transmission, IPV use may not prevent transmission, and failed outbreak response efforts may lead to the need to restart OPV [14].

## What if we need to restart OPV?

Current efforts seek to identify potentially better OPV vaccine virus candidates that offer lower risks of VAPP and VDPVs [19]. In addition, the GPEI will need to work with the current OPV manufacturers to maintain some capacity to restart OPV production quickly if needed. While we hope to never need it, we should develop a plan that considers all of the issues associated with OPV restart for one or more poliovirus serotypes.

## Conclusion & future perspective

The polio endgame requires continued active management to reduce the probability and consequences of potential reintroduction of live polioviruses. Polio eradication promises significant health and economic benefits [10,20], and maximizing these depends on making the best risk management choices and investments now and into the future.

## open access

This work is licensed under the AttributionNonCommercial-NoDerivatives 4.0 Unported License. To view a copy of this license, visit http://creativecommons.org/ licenses/by-nc-nd/4.0/
